# Recent Advances in Biosensor Technologies for Meat Production Chain

**DOI:** 10.3390/foods14050744

**Published:** 2025-02-22

**Authors:** Ivan Nastasijevic, Ivana Kundacina, Stefan Jaric, Zoran Pavlovic, Marko Radovic, Vasa Radonic

**Affiliations:** 1 Institute of Meat Hygiene and Technology, Kacanskog 13, 11000 Belgrade, Serbia; 2 University of Novi Sad, Biosense Institute, Dr Zorana Djindjica 1a, 21000 Novi Sad, Serbia; ivana.kundacina@biosense.rs (I.K.); sjaric@biosense.rs (S.J.); zoran.pavlovic@biosense.rs (Z.P.); marrad@biosense.rs (M.R.); vasarad@biosense.rs (V.R.)

**Keywords:** biosensors, meat chain, pathogen detection, contaminants, food safety, quality assurance, traceability

## Abstract

Biosensors are innovative and cost-effective analytical devices that integrate biological recognition elements (bioreceptors) with transducers to detect specific substances (biomolecules), providing a high sensitivity and specificity for the rapid and accurate point-of-care (POC) quantitative detection of selected biomolecules. In the meat production chain, their application has gained attention due to the increasing demand for enhanced food safety, quality assurance, food fraud detection, and regulatory compliance. Biosensors can detect foodborne pathogens (*Salmonella*, *Campylobacter*, Shiga-toxin-producing *E. coli*/STEC, *L. monocytogenes*, etc.), spoilage bacteria and indicators, contaminants (pesticides, dioxins, and mycotoxins), antibiotics, antimicrobial resistance genes, hormones (growth promoters and stress hormones), and metabolites (acute-phase proteins as inflammation markers) at different modules along the meat chain, from livestock farming to packaging in the farm-to-fork (F2F) continuum. By providing real-time data from the meat chain, biosensors enable early interventions, reducing the health risks (foodborne outbreaks) associated with contaminated meat/meat products or sub-standard meat products. Recent advancements in micro- and nanotechnology, microfluidics, and wireless communication have further enhanced the sensitivity, specificity, portability, and automation of biosensors, making them suitable for on-site field applications. The integration of biosensors with blockchain and Internet of Things (IoT) systems allows for acquired data integration and management, while their integration with artificial intelligence (AI) and machine learning (ML) enables rapid data processing, analytics, and input for risk assessment by competent authorities. This promotes transparency and traceability within the meat chain, fostering consumer trust and industry accountability. Despite biosensors’ promising potential, challenges such as scalability, reliability associated with the complexity of meat matrices, and regulatory approval are still the main challenges. This review provides a broad overview of the most relevant aspects of current state-of-the-art biosensors’ development, challenges, and opportunities for prospective applications and their regular use in meat safety and quality monitoring, clarifying further perspectives.

## 1. Introduction

Biosensors represent a promising and potent tool for enhancing animal health and welfare, as well as food safety, by providing early information due to the possibility for the rapid and on-site detection of various hazards in the food production chain [1]. The meat production chain is complex, involving multiple stages along the F2F continuum. Other issues closely intersecting the meat chain are related to the environmental impacts of intensive livestock production (e.g., on arable land/soil, water, forests, and the atmosphere) and challenges in finding the appropriate and most effective solutions to adopt policies for the management of production systems that are sustainable, economically justified, and ethically accepted, which provide continuous meat/meat products supply on a global scale. The optimization of livestock management systems requires the integration of a variety of data and acquired knowledge on the environment, agricultural practices, biotechnology, animal husbandry, nutrition and behavior, welfare, veterinary medicine, slaughter and meat processing, distribution, and retail with modern electronic systems/devices able to detect specific biomarkers and translate them into readable signals [2,3]. Such sensing systems can play an important role in the transformation of the meat supply chain by improving animal health, productivity, and the food safety of meat and meat products, ultimately providing a higher level of consumer protection.

**Figure 1:**
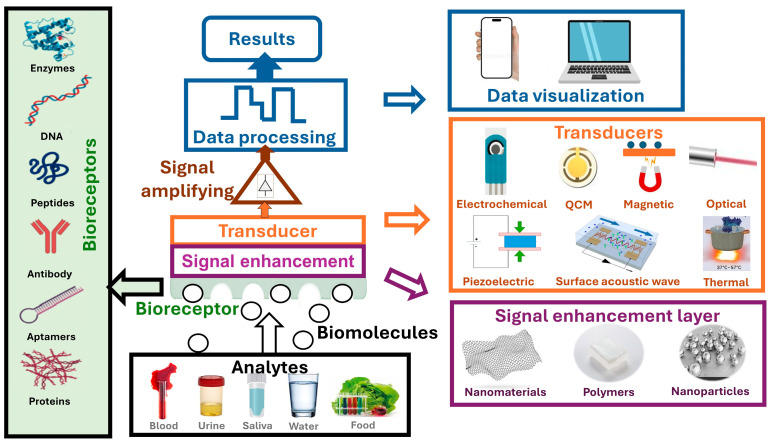
Common components of a biosensor and its working principles used to detect various biomolecules from different analytes.

Biosensors are analytical devices that combine a biological component (such as enzymes, antibodies, nucleic acids (DNA and RNA), aptamers, or whole cells) with a transducer and detector system (Figure 1) to rapidly and cost-effectively detect specific substances from analytes with a high sensitivity and specificity [4,5,6]. The biological component (bioreceptor) incorporated within the biosensor interacts with the analyte on the transducer, which produces a measurable signal that is then converted into a user-understandable, mostly quantifiable output.

**Figure 2:**
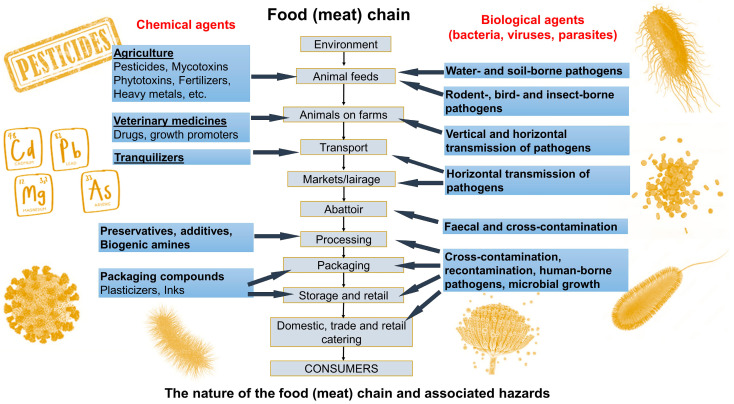
Example of the meat chain structure and associated hazards.

Different foodborne hazards can enter the meat chain at multiple points (Figure 2), carrying potential risks that can compromise food safety and quality [3,7,8]. Animal health and welfare monitoring on-farm (pre-harvest) is based on concepts such as the Heard Health Surveillance Program (HHSP), while food safety hazards in later stages of the meat chain, such as slaughter (harvest), meat processing, distribution, and retail (post-harvest), are controlled by Hazard Analysis and Critical Control Points (HACCPs). These concepts are risk-based, aiming to identify, detect, and prevent/control food-producing-animal-based food safety hazards in a proactive way during the production process, before such hazards can contaminate final food products. Such a proactive approach requires real-time and early information on animal health and food safety hazards so that adequate corrective measures can be applied to eliminate or reduce such hazards before entering later stages along the meat production chain. Current practices do not provide adequate technical support (on-site detection) to fulfill early and prompt reactions, since they are based on the collection of samples, e.g., blood, feces, slurry (on the farm) or swabs from carcasses, meat juice, and lymph nodes (at slaughterhouse), and transportation in cold chain environments (cool bin) to a central laboratory for time-consuming and labor-intensive analysis (e.g., culturing, Enzyme-linked Immunosorbent Assay—ELISA, Polymerase Chain Reaction—PCR, and High-Performance Liquid Chromatography—HPLC), which also requires expensive equipment, a specially designated space, and highly trained laboratory personnel. For example, the average time for obtaining the ELISA and PCR or PCR-HPLC assay results is within 24 h at best [9,10], while for culturing techniques (by internationally recognized standards, such as by the International Standard Organization/ISO), which are considered to be the ‘gold standard’ for the sensitive and specific detection of foodborne pathogens, the results are available after 5 days (such as in the case of *Salmonella* detection; ISO 6579 [11]) or 7 days (for *Listeria monocytogenes* detection; ISO 11290 [12]). On the other hand, biosensors have emerged as an innovative technology offering a low-cost solution for in-field detection, aiming to meet the Real-time connectivity, Ease of specimen collection, Affordable, Sensitive, Specific, User-friendly, Rapid, Robust, Equipment free, and Deliverable to end users (REASSURED) criteria [13,14]. Biosensors can enable a rapid response time for obtaining accurate results and quantitative detection, with an average read-out time of up to 30 min [15,16], which enables timely reactions in applying corrective measures within HHSP and/or HACCPs, thus significantly improving the performance of such risk-based animal health and food safety management systems. Therefore, the major advantages of the use of biosensors over ‘traditional’ methods’ (culture techniques, ELISA, and PCR), which are time-consuming and require expensive equipment, adequate laboratory space, and highly trained personnel, is their user-friendly mode. This relates to their on-site field application, providing early diagnostics and facilitating food chain information (FCI) flow across the meat chain due to their capability to provide (i) rapid detection—an approximate output of up to 30 min, allowing for real-time or near-real-time monitoring; (ii) high sensitivity and specificity—the detection of very low levels of pathogens, contaminants, or spoilage markers, which is crucial for early intervention; and (iii) on-site testing—biosensors as lab-on-a-chip (LOC) and POC devices can be used directly in farm environments, processing plants (slaughterhouse and meat processing), or retail settings, thus reducing the need to send samples to the lab.

Globally, consumers’ perceptions of animal health, animal welfare, and food safety issues have increased in significance over the previous decade, and the demand for proper, real-time, and accurate information on the aforementioned meat safety issues needs to be met for consumers to make informed choices when purchasing preferred meat/meat products [17]. Further, the meat production system is facing the impacts of climate change, as reflected in trends of global temperature increases, precipitation, and wind patterns that are directly or indirectly associated with human activity [18]. Extreme weather events have become more frequent, severe, and unpredictable. These events may jeopardize food safety by changing the ecological patterns and dynamics of different hazards, including foodborne ones, by altering their occurrence, virulence, and distribution, leading to an increased exposure for consumers [19]. For example, a potential association between rising temperatures and increased levels of antimicrobial resistance (AMR) in certain zoonotic food (meat)-borne pathogens has been observed, e.g., *Campylobacter* spp., *Salmonella* spp., *Listeria monocytogenes*, and *E. coli*. Parallel to this, these pathogens are also showing an increased resistance, in particular, to Critically Important Antibiotics (in accordance with the World Health Organization/WHO CIA list) and/or Medically Important Antibiotics (in accordance with the WHO MIA list), thus reducing the efficacy and quality of clinical treatments [20,21,22,23]. The meat production chain is also facing another challenge related to its sustainability due to the environmental impacts of livestock production contributing to anthropogenic greenhouse gas emissions to some extent [19]. Mitigation strategies that include early sensor-based information on animal health and welfare can significantly reduce emissions, enabling the monitoring and optimization of farm animals’ digestion, feed conversion, and a better product yield [24]. In the context of meat safety, biosensors can be effectively used to monitor food safety and quality by detecting foodborne pathogens such as *Salmonella*, *Campylobacter*, *Listeria*, and pathogenic *E. coli*, chemical contaminants such as antibiotics, pesticides, dioxins, heavy metals, and mycotoxins [25], specific biochemical markers that indicate animal health (acute-phase proteins) and welfare (stress hormones) [26,27], meat spoilage assessments by detecting ammonia, biogenic amines, and volatile organic compounds (VOCs) [28], and food authenticity/food fraud (the presence of undeclared species or the dilution of meat with other substances) verification based on species identification [29,30]. Lastly, a new challenge is related to the process control of cell-based (cultivated) meat, which is based on culturing cells isolated from animals followed by processing to produce food products that are comparable to their corresponding animal versions [31]. Potential food safety hazards (fecal-borne pathogens) during cell selection for meat cultivation, production (e.g., *Mycoplasma*), harvesting (biological components, such as growth factors and hormones from animal serum), food processing, and formulation (additives, ingredients, and nutrients) [32] should be also regularly monitored to ensure food safety.

In spite of the number of developed biosensing platforms for the detection of different animal health, animal welfare, food safety, and food quality markers, their application within regulatory frameworks is still a debated issue [33]. The major challenges to be tackled are related to their specificity (although highly sensitive, some biosensors may suffer from cross-reactivity, where they detect non-target substances, leading to false-positive results), costs (the regular use of biosensors can be expensive, which may limit their widespread adoption, especially in smaller operations), and calibration/standardization (ensuring that biosensors are consistently accurate across different batches and environments) [33,34]. The major drivers related to the adoption of biosensors and their regular introduction into meat production systems are related to the following: (i) regulatory compliance, such as food safety standards (stricter food safety regulations are pushing meat producers to adopt technologies like biosensors to ensure compliance) and traceability requirements (demand for greater transparency and traceability in the food chain), (ii) consumer demands, such as quality assurance (with increasing consumer awareness and demand for high-quality, safe meat products are driving the adoption of biosensors) and sustainability (consumers are also pushing for sustainable practices, which biosensors can support by reducing waste and improving efficiency) [35], (iii) technological advancements, such as portability (POC devices based on advances in nanotechnology and microelectronics) [36] and real-time monitoring (improved data analytics and connectivity allow for real-time monitoring and rapid response to potential safety issues) [37], (iv) economic factors, such as cost-effectiveness (since, as the cost of biosensor technology decreases, it becomes a more attractive option for meat producers to improve the safety and efficiency of production processes [31]), (v) sustainability and loss prevention (by allowing for the monitoring of contamination and spoilage, biosensors help to prevent and/or reduce food waste and economic losses) [5], and (vi) globalization, such as international trade (the global nature of the meat industry, with products often crossing multiple borders, needs rigorous safety checks, which biosensors can facilitate) and competitive advantages (companies adopting biosensors may gain a competitive edge by offering safer, higher-quality products) [38].

This review highlights the following: (a) the most relevant and up-to-date aspects of the current state-of-the-art of biosensors’ development and manufacturing, (b) challenges and opportunities for prospective biosensors’ application and regular use in meat safety and quality monitoring, and (c) research and development needs to address these challenges and improve biosensors’ reliability and affordability.

## 2. Materials and Methods

A literature review was conducted by identifying and analyzing articles (research and review scientific papers, technical reports, and guidelines by international organizations) published in the domains of biosensors and sensing systems related to meat safety, meat quality assurance, food fraud, food control, public health, zoonotic foodborne pathogens, antimicrobial resistance, meat chain, meat-producing animals, animal health, animal welfare, veterinary medicine, and detection methods. The searched documents originated from international scientific databases such as the Web of Science, Scopus, Academic Search Complete, IEEE Xplore, PubMed, EBSCO, and CAB Abstracts. The search algorithm included relevant keywords and phrases related to the topic and was based on Boolean operators (AND, OR, and NOT) to combine keywords and narrow down the results. These included terms like “Biosensors AND meat safety”, “Biosensors AND meat quality”, “Biosensors AND food fraud”, “Biosensors AND public health”, “Biosensors AND zoonotic food borne pathogens”, “Biosensors and meat-producing animals”, “Biosensors AND animal health”, “Biosensors AND animal welfare”, “Biosensors AND detection methods”, “Biosensors AND antimicrobial resistance”, “Biosensors AND veterinary medicine”, “Biosensors AND food control”, “Biosensors AND drivers”, “Biosensors AND nanotechnology”, “Biosensors AND manufacturing”, and “Biosensors AND multiplex”. The search was performed for the years between 1998 and 2025. Each source of information was further checked by reading through the titles and abstracts of the search results to assess its relevance and eligibility for the given topic. In total, 1200 publications were retrieved, while 231 publications were selected for the purposes of preparing this review. Once a list of relevant articles was selected, a “snowballing” technique was used to discover the additional literature listed in the initially retrieved articles. The selection criteria to identify relevant articles within the scope of this review were as follows: (1) current state-of-the-art biosensors’ applications in the meat chain and (2) a focus on prospective biosensors’ use in the meat production chain, within the regulatory framework for the monitoring of animal health, animal welfare, and meat safety and quality.

## 3. Overview of Different Biosensor Types

Biosensors in the meat production chain are specialized devices designed to detect biological or chemical changes, enabling the monitoring of meat safety and quality at various stages, including the detection of animal health and welfare and applications in food crime and food fraud control. As shown in Figure 1, biosensors can operate using various detection principles, including optical, piezoelectric, magnetic, acoustic, electrochemical, thermal, and others. The selection of the detection method is influenced primarily by the specific purpose of the biosensor, as well as factors such as the sample type, required specificity, accuracy, and detection limits. Additionally, signal enhancement layers composed of different nanomaterials, nanoparticles, and polymers or their combination can be used to improve sensor performances, resulting in the better binding of biomolecules and an improved specificity or selectivity of the sensor, etc. Biosensor characteristics are also determined by the sensor manufacturing technology itself. In recent years, thanks to the development of modern micro- and nanotechnologies and new materials and microfluidics, a number of new biosensor solutions have been published in the literature, which, in addition to the biosensor element itself, include the integration of these sensors into complex POC and LOC systems, which integrate filtration, separation, reagent mixing, pre-concentration, amplification, and electronic read-out on the same portable device [4,5]. While numerous devices have been developed for applications across the meat chain, this section focuses on the most commonly used transducer techniques, given their widespread applicability and relevance. In this section, both the working principle of these sensors and their fabrication technology are explained, together with a comprehensive overview of the general applications of different biosensor transduction principles throughout the F2F continuum, highlighting their roles in ensuring safety, quality, and traceability in meat production.

### 3.1. Electrochemical Biosensors

Electrochemical (EC) biosensors are a class of analytical device that convert chemical reactions into electrical signals, enabling the detection of specific analytes with a high precision [39,40]. EC biosensors integrate a biological recognition element with an EC transducer electrode to detect targeted analytes. These recognition elements can be ion-selective membranes or biomolecules that interact selectively with the target analyte immobilized on an EC sensor, initiating a biochemical reaction. EC biosensors commonly operate based on one of the following three detection techniques: amperometry (measures current changes resulting from redox reactions involving the analyte), voltammetry (monitors the potential differences between electrodes caused by ionic interactions), or impedimetric detection (variations in the electrical impedance of the sensor interface upon analyte binding). These approaches enable highly sensitive measurements, even at trace levels. EC biosensors are highly valued for their sensitivity and rapid response, making them indispensable tools in food safety applications. The specificity of EC biosensors is derived mainly from the biological recognition element. Enzyme-based biosensors, for instance, utilize catalytic reactions to produce electroactive species detected amperometrically [40]. Immunosensors leverage antibody–antigen binding to trigger changes in impedance or potential, while DNA biosensors detect hybridization events by measuring electron transfer [39]. Furthermore, EC sensors are advantageous from a manufacturing perspective due to their relatively simple and cost-effective fabrication processes, which allow for mass production using techniques like screen printing, inkjet printing, and sputtering. Additionally, their miniaturization potential enables the development of planar, portable, and disposable sensors, enhancing their accessibility and ease of use in various applications. However, they can be sensitive to interference from complex sample matrices and may have a limited stability over prolonged use.

Recent advancements in nanotechnology have further enhanced EC biosensors’ detection capabilities. Nanomaterials, such as graphene, carbon nanotubes, and metallic nanoparticles, are often incorporated into the sensor design to enhance the signal on the transducer [41,42]. These materials provide a high surface area, enhance electron transfer rates, and improve signal-to-noise ratios, enabling the detection of analytes at extremely low concentrations.

In the food industry, including the meat production chain, EC biosensors can be critical for ensuring the safety and quality of the raw material and final product. They are widely used to detect pathogens, toxins, allergens, and chemical residues in meat/meat products [43,44]. Nowadays, portable EC sensors have gained popularity for real-time monitoring in food production, storage, and distribution, ensuring compliance with regulatory standards [45]. Furthermore, enzyme-based electrochemical biosensors are being increasingly used to monitor freshness indicators, such as biogenic amines in meat products [46], while continuous quality monitoring throughout the supply chain can be realized using biosensors on smart packaging [47]. Moreover, EC biosensors have been developed for the detection of nutritional value, providing information about adulteration [48], nanomaterial-based EC sensors have been developed for the detection of antibiotics in pork and chicken meat [49], and EC biosensors have been developed for pathogens with magnetic-labeling nanoparticles for the detection of *E. coli* [50].

### 3.2. Optical Biosensors

Optical biosensors offer a high sensitivity and the ability to detect analytes without the need for labels, making them ideal for real-time, in situ analyses in various applications such as food safety and environmental monitoring [51]. They use light-based detection methods, such as color changes, fluorescence, surface plasmon resonance, and refractive index changes, to monitor biological interactions. The interaction of light with biomolecules in the analyte is generally expressed with changes in reflection, transmission, and absorption, which enable the development of various optical biosensors. Among the simplest optical biosensors are colorimetric biosensors. Their detection principle is directly related to changes in color, which can be observed by the naked eye or with the detector [52,53]. This is also one of the oldest detection methods used for routine food analysis, since the freshness and quality of the food can be directly monitored with color changes.

Another class of optical biosensors encompasses fluorescence biosensors, which use fluorescence to detect biological molecules, such as pathogens, drugs, and toxins [54]. Fluorescence biosensors work by binding the target molecule to a fluorescently labeled probe, which results in changes in the fluorescence intensity or emission wavelength of the probe. The essential components of fluorescence biosensors are an excitation light source (such as light-emitting diodes/LEDs or lasers), fluorophore molecules that mark target biomolecules, and a photodetector that captures the fluorescence intensity and spectrum. The major advantage of these fluorescence biosensors is their high sensitivity and selectivity, allowing for the detection of low concentrations of target analytes with minimal interference. Additionally, fluorescence biosensors can be used for fast, real-time monitoring in complex samples without the need for extensive sample preparation or labeling.

Photonic biosensors utilize light-based technologies, such as interference, diffraction, and resonance, to detect biological interactions and analytes with a high sensitivity and precision. Most photonic applications are in the visible and near-infrared light ranges, albeit spanning all technological uses of light over the whole spectrum [55]. Photonic biosensors can be fabricated on a substrate with a low refractive index and low thickness, using silica or polymer materials, or in the form of optical fibers. Most familiar types of photonic sensors are based on optical fiber technology, where optical fibers are used to detect biological molecules [56]. These sensors typically consist of a fiber-optic waveguide, a sensing region, and a detection system.

Surface plasmon resonance (SPR) is a phenomenon in which light interacts with a metal film or metallic nanoparticles to produce a strong confinement of the electromagnetic field intensity. This confinement enhances the interaction between light and the target molecules, making the measurement more sensitive. An SPR biosensor works by shining light onto a metal film or nanoparticle and measuring the angle of minimum reflection, also known as the angle of maximum absorption. The fabrication of SPR sensors can be complex and challenging, as it requires the precise control of nano-scale metal structures on a substrate. These metal structures must be of the correct size, shape, and orientation to allow for the efficient excitation of surface plasmons, which is the key to the high sensitivity of SPR sensors, but also the main disadvantage of this type of biosensor. One of the key advantages of SPR biosensors is their high sensitivity, which enables the detection of trace amounts of biological molecules. SPR has found a wide range of applications, including detection in animal healthcare, food inspection, and allergen detection [57,58].

Surface-Enhanced Raman Spectroscopy (SERS) sensors are a type of photonic biosensor that utilizes the unique optical properties of nanostructured materials to enhance the Raman scattering signal from analytes. SERS is a highly sensitive analytical technique that can detect trace amounts of chemical species, making it an attractive technology for various sensing applications, including in the field of animal welfare and healthcare [59,60]. Since spontaneous (normal) Raman scattering is typically very weak, Surface-Enhanced Raman Spectroscopy employs a special technique to enhance the Raman scattering by molecules adsorbed on a specific medium or interface to improve the sensitivity. The key advantages of SERS sensors are their high sensitivity and versatility. SERS can be applied to a wide range of substrates, including surfaces, fibers, and nanoparticles, and can be integrated with various imaging and sensing systems, such as optical microscopy and spectroscopy.

Optical biosensors offer a high sensitivity, fast response times, and label-free detection, making them highly effective for real-time monitoring. Their fabrication involves advanced techniques such as photolithography, electron-beam lithography, and nanoimprinting, which allow for precise control over sensor properties, but can increase production complexity and cost. In addition, thin-film deposition techniques such as sputtering, evaporation, and chemical vapor deposition are also commonly used to create biosensitive optical layers with tailored properties. While their miniaturization enables portability, their integration with optical components can be challenging, requiring specialized equipment for signal detection and processing. Moreover, environmental factors such as temperature fluctuations and light interference can affect their stability and accuracy.

In summary, colorimetric sensors are widely used in the meat industry for the rapid and cost-effective detection of spoilage, contaminants, and quality indicators such as pH, ammonia, and biogenic amines [61,62,63]. Their main advantages include easy visual interpretation without the need for complex instrumentation, real-time monitoring capabilities, and the potential for integration into smart packaging for the continuous assessment of meat freshness. Some additional examples include SPR sensors used for detecting foodborne pathogens such as *Salmonella* and *E. coli* by monitoring refractive index changes upon biomolecular binding [64,65], fluorescence-based biosensors for the detection of meat freshness markers [66], and Raman-spectroscopy-based sensors to provide molecular fingerprinting for the identification of adulterants, such as the presence of unauthorized additives or the mislabeling of meat species or mycotoxins [67,68]. Additionally, optical fiber biosensors have also been employed for detecting antibiotic residues in meat, offering a high sensitivity and real-time analysis [69], while different types of optical sensors have been used in meat authentication (the detection of meat species), thus helping to prevent food fraud [30].

### 3.3. Field-Effect Transistor (FET)-Based Biosensors

An FET-based biosensor is a type of label-free biosensor where the bio-interaction is directly converted into an electrical signal, measurable by a suitable instrument [70,71,72]. Speaking traditionally, FETs are semiconductor-based devices that consist of the following three main terminals (electrodes): source, drain, and gate. The current flow in an FET is controlled by an electric field, established by the gate potential, and such flow takes place in an FET channel, which is the most important part of FET-type biosensors. Additionally, the fourth terminal in an FET is a body or substrate, which is needed to bias the transistor into operation [73]. There are numerous types of FETs, classified mainly by the nature of the channel, material used, or the physical principle of the operation. Biosensors that work on the FET principle (BioFET—biologically sensitive FET) demand slight changes in the standard structure of an FET. Namely, the most efforts are directed to channel modification in order to enable unprecedented performances in sensing that are not achieved with some traditional detection techniques. The incorporation of a wide spectrum of nanomaterials has opened the door to increased FET-based biosensor interest and research, especially wuth two-dimensional (2D) materials [74,75,76]. These 2D-FETs can operate in the following two modes concerning gate orientation: back-gate (solid-gate) or top-gate (liquid-gate). For biosensing, a top-gate regime is mostly used nowadays. Namely, the gate electrode is often comparable to the standard reference electrode in an electrochemical three-electrode system and is immersed in the electrolyte of interest or colinearly fabricated on the body of the FET. In this configuration, an insulation layer is formed upon the application of gate voltage in the form of a so-called electrical double layer, which serves as a capacitor at the solid/liquid interface with a thickness of several nanometers and a capacitance of several orders of magnitude higher than induced back-gate capacitance. Likewise, the liquid-gate configuration makes the conductivity of the channel more sensitive to specific interactions [77]. Two-dimensional materials are very sensitive to charge redistribution in the vicinity of their surface; with integration into an FET arrangement, the electrical properties are very susceptible to such redistribution. Consequently, channel materials are tuned with specific biorecognition elements to make biosensors specific to certain target molecules [78].

FET-based biosensors offer an exceptional sensitivity due to their ability to detect minute changes in charge distribution upon target binding, making them highly effective for real-time analysis. Their fabrication involves advanced semiconductor processing techniques, such as nanolithography and the deposition of nanomaterials like graphene or silicon nanowires, which enhance performance but increase production complexity and cost. While FET biosensors provide rapid, label-free detection with low sample volume requirements, they can be prone to signal drift, interference from ionic strength variations, and stability issues over time. Despite these challenges, their miniaturization potential, compatibility with integrated circuits, and low power consumption make them suitable for portable and high-performance biosensing applications.

Nowadays, the application of portable sensing devices to track animal welfare and health parameters is a practice [79]. However, using wearable FET devices in animal health monitoring has not been well exploited. Although FET-based biosensors with various functional nanomaterials for a high sensitivity and reproducibility show an excellent potential for the detection of different biomolecules in other applications, only several applications in the meat chain are available in the literature. For example, aptamer-based FET biosensors with a carbon nanotube were developed to detect pathogenic bacteria [80] and aflatoxin B1 [81]. In addition, an FET e-nose biosensor was developed for meat product freshness control [82].

### 3.4. Piezoelectric Biosensors

Piezoelectric sensors offer a high sensitivity, fast response, and the ability to detect very small changes in mass, making them ideal for biosensor applications. The manufacturing technology of piezoelectric sensors is relatively simple and can use materials such as quartz, allowing for integration with other systems and reduced production costs. A growing interest in the field of biosensors is devoted to the development of quartz crystal microbalance (QCM) biosensors. This type of biosensor utilizes quartz crystal resonator technology to detect and analyze biomolecules based on changes in the mass of a sensing layer due to the binding of analytes. This highly sensitive and label-free approach offers real-time detection using a portable system with basic data analysis. QCM operation is based on the piezoelectric effect in a quartz crystal, where changes in mass on the crystal surface cause shifts in its resonant frequency. When a substance adsorbs onto the crystal surface, the added mass alters the vibration frequency of the crystal. This frequency change is directly proportional to the amount of mass. The proposed QCM solutions are usually based on a gold substrate that is modified with a biorecognition element like immobilized antibodies, nucleic acid probes, or synthetic molecularly imprinted polymers (MIPs), which are also known as artificial antibodies, for specific detection in a complex analyte [83]. QSM biosensors are typically fabricated using microfabrication techniques such as thin-film deposition, photolithography, screen printing, and electroplating to create the quartz crystal, electrodes, and sensing layers. Although QCM can detect very small changes in mass, piezoelectric sensors can be susceptible to accuracy degradation in environments with significant temperature or humidity fluctuations, as these factors can affect frequency changes. Additionally, the specificity of piezoelectric sensors may be limited in cases where differentiation between similar molecules or substances is required.

Recent studies in the field of QCM biosensors have focused on applying nanomaterials for sensing and improving sensors’ sensitivity [84,85]. In the meat industry, QCM sensors functionalized with aptamers have been used to detect pathogens in meat [86], fever viruses using an MIP layer [87], for drug detection [88], or to detect meat adulteration [89]. While QCM biosensors have shown promise in the detection of pathogens and other quality indicators, the sensitivity of QCM biosensors can be affected by the matrix in which the analyte is present. Components such as fats and proteins can interfere with the binding of the analyte to the sensing layer, resulting in a reduced sensitivity and accuracy.

### 3.5. Surface Acoustic Wave Biosensors

Surface acoustic wave (SAW) biosensors utilize the interaction between an electrical signal and surface acoustic waves to detect changes in the environment. SAW biosensors operate by generating acoustic waves on the surface of a piezoelectric material, where any mass changes or interactions on the surface affect the wave’s velocity or amplitude. These changes are then measured and correlated with the presence or concentration of the target analyte, offering a high sensitivity and real-time detection capabilities. These sensors have gained popularity due to their high sensitivity and small size, making them an attractive choice for a wide range of applications [90,91]. SAW sensors are fabricated using piezoelectric materials like quartz or lithium niobate, where interdigital transducers are patterned onto the surface using photolithography or thin-film deposition techniques. The fabrication process involves precise control of the electrode patterns and the integration of the sensing layers to ensure a high sensitivity and stability. Ongoing research efforts are focused on improving the performance of SAW sensors, including increasing their sensitivity and expanding their range of detectable analytes. The application of these sensors in the farm-to-fork continuum is still rare. SAW sensors have been developed to evaluate chicken meat storage time [92], for haptoglobin detection in unpurified meat juice from slaughtered pigs [93], and for pathogen detection [94].

### 3.6. Magnetic Relaxation Switching Biosensors

Magnetic relaxation switching (MRS) biosensors are a promising technology that has gained significant attention in recent years for its ability to detect target biomolecules with a high sensitivity and selectivity [95]. MRS biosensors use functionalized magnetic nanoparticles that bind to the target biomolecules and measure changes in the magnetic field. These changes are detected using an external magnetic field, providing a highly sensitive and quantitative read-out of the target biomolecule concentration. Detection devices for MRS biosensors typically include highly sensitive magnetometers, such as superconducting quantum interference devices or vibrating sample magnetometers, which measure the changes in the magnetic field caused by the interaction of magnetic nanoparticles with the target analyte. This technology offers a high sensitivity and selectivity due to the unique magnetic properties of the nanoparticles, but its fabrication process can be complex, requiring precise control over nanoparticle synthesis, functionalization, and sensor integration. While MRS biosensors provide excellent detection capabilities in complex samples, their manufacturing complexity and the need for specialized equipment can limit their widespread application. Recently proposed MRS biosensors in the literature demonstrate very sensitive detection performances for low concentrations of pathogens in meat samples [96,97], as well as forbidden substances and residues related to drugs for animal treatments [98], and toxin detection [99].

## 4. Application of Biosensors in the Meat Chain Continuum

**Figure 3:**
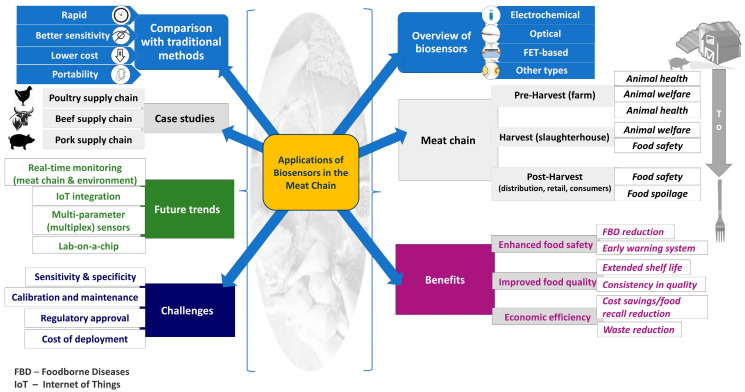
Current and future perspectives of biosensors’ application in the meat supply chain.

Biosensors play a crucial role in enhancing safety, quality, and efficiency within the meat supply chain. The application of biosensors in the meat chain spans all modules in the F2F framework, ensuring that meat products are safe, of a high quality, and traceable throughout the entire continuum. Multiple of their applications in the meat production chain are presented in Figure 3.

### 4.1. Animal Health

In recent years, biosensors have been increasingly applied in the monitoring of animal health and welfare, including reproductive and nutrition status. As biosensors can provide real-time and reliable data on various biomarkers that indicate the general and specific health statuses of farm animals [2], they represent a powerful tool to help in the early detection of diseases, as well as the management of chronic conditions. Examples of the applications of such biosensors in the animal health sector are given in the sections below.

#### 4.1.1. Detection of Disease Markers

*Blood Glucose Levels.* Biosensors enable continuous glucose monitors (CGMs) and can be used for diabetic animals or those at risk of metabolic disorders (e.g., cetosis) [100,101]. Different enzymatic sensors have been proposed in the literature [102], while some commercial solutions are already available on the market for veterinary use [103]. Early information on blood glucose levels can help in adjusting nutrition regimes in a timely manner, as well as the farm environment to prevent the occurrence of metabolic disorders.

*Hormonal Levels.* Cortisol and/or Chromogranin A (CgA) sensors can monitor the stress levels in animals, which can indicate welfare issues or the presence of disease [2,27,104]. Further, information on the levels of specific stress hormones can also provide insights into farm husbandry practices and valuable data for the prediction of meat quality traits in further processing, also by using non-invasive methods [104], such as the detection of glucocorticoids using non-invasive sample materials like saliva, excreta, milk, hair/feathers, and eggs [105]. Recently, an EC immunosensor with antibodies and aptamers as its bioreceptors was proposed to monitor cortisol levels [106,107], including different optical sensors that can be efficiently applied in animal welfare for the tracking of stress levels by on-site measurements of stress-related metabolite levels or sensors for the detection of hormones such as cortisol molecules at concentrations as low as 2 ng/m, as well as other hormones important for the reproductive cycle (progesterone, testosterone, and 17β-estradiol) in urine [108,109]. Further, EC biosensors have been also used for the detection of animal hormones in food, including meat [110]. A comprehensive review regarding the detection of various metabolites and biomarkers in cattle was published, outlining biosensors for the diagnosis of noncompliant pH, dark cutting beef predisposition, and welfare [111]. Wearable sensors have also been extensively used for monitoring animal welfare in disease detection [112], where, for example, tattoo-based biosensors were utilized to monitor the metabolites in interstitial fluid, such as pH, glucose, and albumin concentrations, using minimally invasive, injectable skin biosensors [113].

*Inflammatory Markers.* Biosensors can measure the levels of acute-phase proteins (e.g., C-Reactive Protein or haptoglobin), which gives information on inflammation, infection, and injury, as well as cytokines (TNF-alpha and IL-6), which are indicators of immune response and inflammation in animals. Namely, poorly managed farm biosecurity may lead to the occurrence of different acute and chronic disease conditions in food-producing animals (e.g., cattle, pigs, and poultry) [114] induced by a variety of pathogens of viral and bacterial origin, provoking a range of respiratory, gastrointestinal, skin, and udder infections (e.g., rotavirus infections, bovine respiratory disease, bovine viral diarrhea, avian, pig of bovine influenza, mycoplasmosis, salmonellosis, colibacillosis, staphylococcosis, mastitis, etc.) [115,116,117]. Early information on specific acute-phase proteins (APPs) may contribute to assessing the general health status of animals, including unnoticed subclinical conditions, thus preventing the further aggravation of disease conditions and/or carrying out timely therapeutic protocols [5,104]. For example, a biosensor detecting a specific APP such as haptoglobin (Hp) can provide real-time information on mastitis in milking cows [118], inflammation/infection or trauma in beef and dairy herds [119], and the future development of biosensors able to detect Pig-Major Acute Proteins (Pig-MAPs) important for revealing infection with H3N2 swine influenza virus or other inflammatory conditions in pigs [114,120,121].

*Metabolic Markers.* The monitoring of selected markers associated with the metabolism of animals can be conducted by biosensors. For example, biosensors may provide useful information for the diagnosis of disease by the detection of VOCs from animals’ breath, blood, faeces, skin, urine, and vaginal fluids [2]. Breath metabolites are composed of gases (e.g., hydrogen and methane) and fatty acids, which all can be used as specific biomarkers for the detection of certain metabolic and pathologic processes. For example, a higher level of glucose in the blood is detected by the presence of specific VOCs, e.g., ketones, ethanol, and methanol [122]. In livestock, these biosensors can accurately identify bovine respiratory diseases (BRDs) [123], brucellosis [124], bovine tuberculosis [125], Johne’s diseases [126], ketoacidosis [127], and even Foot and Mouth (FMD) disease [128]. Other biosensors enable the salivary detection of metabolites, such as the level of uric acid, high levels of which may be correlated with metabolic syndrome, renal syndrome, or physical stress [129] and provide the detection of perspiration metabolites such as the concentrations of sodium and lactate in sweat, also giving information about animal welfare (e.g., physical stress) [130].

*Disease-associated pathogens*. There have been recent developments in biosensors able to detect specific biomarkers from animals’ fluids and tissues, providing valuable input needed for diagnostic procedures for respiratory, gastrointestinal, and other diseases.

*For cattle,* biosensors have been developed as a diagnostic method for bovine respiratory disease (BRD), with a high sensitivity and specificity to the anti-IgE present in commercial anti-BHV_1 (Bovine Herpes Virus-1/BHV-1, the wild-type virus cause of BRD) and in real serum samples from cattle [117,131]. Another sensor was developed to detect virus-provoking bovine viral diarrhea (BVD) from a cattle serum sample, with a very short detection time of only 8 min and a limit of detection (LOD) of 103 CCID/mL [116]. Based on the SPR phenomenon, a biosensor able to detect bovine leukemia virus (BLV), a causative agent of Enzootic Bovine Leucosis (EBL), was developed [132], as well as a biosensor for FMD detection, which included a lateral flow immune-chromatographic platform for the detection of antibodies against FMD proteins [128]. An in-field aptamer-based biosensor was developed for the detection of bovine mycoplasma *(M. bovis*) [133], a disease severely affecting the health of cattle and provoking bronchopneumonia, mastitis, and arthritis. Further, an online sensing system enabling an automated California Mastitis Test (CMT) in milk has been developed [134], as well as a biosensor for the detection of mastitis based on acute-phase proteins (haptoglobin) [135]. Further, a biosensor for the detection of prion protein (PrPC)-causing Bovine Spongiform Encephalopathy (BSE) and Creutzfeldt–Jakob disease in humans and scrapie in sheep has been developed [136], as well as a sensor for the detection of *Campylobacter* in dairy livestock, based on magnetic beads functionalized with a *C. jejuni* aptamer, which uses magnetic separation to isolate and enrich *C. jejuni* from samples, with a detection range of 10–10^7^ colony-forming units (CFU)/mL and an LOD of 3 CFU/mL [137].

*For pigs,* different sensing systems are available, such as a quartz crystal immunosensor for the detection of African Swine Fever virus (ASFV) developed in 1998 using diluted pig sera samples [138], as well as a nanoplasmonic biosensor involving p30-protein-specific label-free integration into standard 96-well plates, able to detect ASFV in 20 min [139], which was proven in an in vitro environment, and colorimetric sensors or aptamer-based sensors for the detection of porcine reproductive and respiratory syndrome virus (PRRSV), respectively [140,141]. This is an important disease occurring on large-scale commercial pig-fattening farms that causes serious impacts on livestock farms worldwide. In addition, a range of photonic (optical) integrated-circuit (PIC) biosensors based on antibody (monoclonal)–antigen reactions were developed within the SWINOSTICS project—Swine diseases field diagnostics toolbox (https://cordis.europa.eu/project/id/771649/results, accessed on 6 January 2025) for the multiplex detection of the six most important endemic and emerging viruses occurring in the pork production chain, including ASFV, Classical Swine Fever Virus (CSFV), PRRSV, Porcine Parvovirus (PPV), porcine circovirus 2 (PCV2), and Swine Influenza Virus A (SIV) [142,143], as well as a photonic sensor for the detection of porcine circovirus 2 (PCV2)-causing porcine circovirus-associated disease (PCVAD) associated with attacks on lymphoid tissues and consequent immunosuppression in pigs [144].

*For poultry,* an impedance immunosensor based on the detection of avian influenza (bird flu)-associated immobilized H5N1 antibodies, providing an LOD of 10^3^ EID_50_/_mL_ (EID50: 50% Egg Infective Dose), was developed [115], as well as a biosensor for detecting and differentiating between avian and human influenza viruses [145]. The importance of the regular and wide-based application of such biosensors can be crucial in the monitoring and early detection of avian influenza, not only in poultry flocks, but also in dairy cattle, taking into consideration the recent first-ever recorded transfer of bird flu to cattle, provoking a multistate outbreak of Highly Pathogenic Avian Influenza (HPAI). This disease (H5N1) in dairy cows was recently reported in the USA [146]. A biosensor containing mammalian cells as sensing elements able to detect enterotoxins of *Clostridium perfringens* (A, B, C, D, E, NetB, and CnaA) causing necrotic enteritis in broiler chickens, which is associated with significant economic losses in the global poultry industry due to high mortality rates, as well as provoking foodborne disease in humans due to the sporulation of pathogens and the development of toxins, has been proposed [147]. Further, a three-mode biosensor with a ratiometric design (electrochemical/colorimetric and electrochemical/photothermal) based on DNA-driven magnetic beads (DMBs) has been developed to detect *C. perfringens* with a very low LOD of only 0.26 and 0.27 lg/CFU in samples in real operational environments, enabling its use in food safety and environmental monitoring [148]. Sensors based on SPR technology, which can accurately detect antibiotic residues (e.g., fluoroquinolones and sulfonamides) in chicken muscle and blood serum, are also available [149]. Although confirmatory methods for antibiotic residues depend on liquid chromatography–mass spectrometry (LC-MS) or a combination of liquid chromatography and an ultra-violet detector (LC-UV) to determine the exact concentration of an analyte, biosensors can be effectively used for screening purposes (semi-quantitative measurements) [150] and serve as a very practical solution when there is a need for the large-scale detection of antibiotic residues in animals in the farm-to-slaughterhouse continuum [149].

#### 4.1.2. Monitoring Reproductive Health

*Hormone Levels.* Sensors can detect the levels of hormones like progesterone and estrogen, which are critical in monitoring reproductive cycles and identifying fertility issues. The reproductive management of dairy herds is of great importance for the dairy industry, with missed estrus detection representing a main cause of economic losses. In the early stages, a biosensor using an enzyme immunoassay format for molecular recognition was developed with a read-out time of 8 min [151]. The gold standard is the detection of progesterone, a hormone important for the reproductive management of dairy cows, based on antibodies, biosensors for which are expensive and often difficult to procure on the international market. In response to this, an affordable transcription-factor-based sensor has been developed in a portable paper-fluidic format to serve the accurate and rapid detection of progesterone [152]. Another solution is a microbially derived biosensor as an affordable, real-time sensor device able to detect progesterone in urine [153]. In addition, a fungal biosensor for the detection of estrogen activity in cows and pregnancy diagnosis, based on a modified strain of the filamentous fungus *Aspergillus nidulans*, was developed, after successful validation with blood, urine, feces, milk, and saliva; it had a specificity of 100% and a sensitivity of more than 97% in milk, urine, and feces [154]. Other examples are biosensors for the detection of mycotoxins in cow’s milk, such as bioluminescent whole-cell biosensors for the detection of zearalenone family mycotoxins, allowing for detection in less than 3 h, based on the fat content in milk, providing an excellent screening device [155]. Other developed ‘mycotoxin biosensors’ based on nanomaterials for the determination of mycotoxins such as endocrine disruptors (EDMs) are also available [156], such as aptamer-based ones with LODs of 0.93 pg/mL [157], 0.17 pg/mL [158], and 2 pg/mL [159], as well as an antibody-based sensor with an LOD of 0.1 pM [160].

*Estrus detection.* The detection of estrus in animals is a crucial issue of high economic importance to farmers in livestock management to optimize reproductive efficiency and productivity. Standard sensor solutions are based on monitoring vaginal conductivity and temperature, while wearable solutions placed in the ventral tail to acquire data such as surface temperature, behavior, and ovulation can also be used [161]. Unfortunately, these solutions can only be confirmed at a rate of 50–60%, which is lower than that obtained using a biosensor. Therefore, a metal oxide electronic nose biosensor was developed to detect estrus based on odor release from the perineal headspace in dairy cattle by direct sampling [162], as well as a biosensor to track the bovine estrous cycle by online measurements of milk progesterone [163].

*Sperm Quality*. Animal sperm quality assessment is crucial for reproductive success in livestock and wildlife management. A microfluidic biosensor with a portable microscopic imaging system was designed to predict the reproductive ability of livestock [164]. Although methods based on counting and micro-imaging systems have demonstrated a high accuracy in detecting sperm survival rates and a fast detection time [165], recent advancements have led to the development of biosensors that evaluate sperm parameters such as motility, viability, and morphology, which are important markers in breeding programs.

### 4.2. Animal Welfare

The application of biosensors enables the early detection of animal welfare issues, including the detection of stress (impact on health and meat safety and quality), such as biosensors able to detect cortisol levels in animal blood (serum), saliva, or urine [106,107,109], as described above. This ensures that animals are raised on farms, transported to livestock markets or slaughterhouses, and processed (slaughter and dressing) under conditions that minimize stress and suffering. These technologies facilitate humane handling practices and support regulatory compliance and consumer transparency. As biosensor technology continues to advance, its role in promoting higher animal welfare standards in the meat industry is likely to grow, benefiting both animals and the industry. Examples of biosensor applications in animal welfare monitoring are given below.

#### 4.2.1. Behavioral, Physiological, and Nutrition Status Monitoring

*Animal Interstitial Fluid.* The recent emergence of biosensors has enabled the detection of internal physiological factors as a component of strategies to improve animal welfare. Such an approach is dependent on the monitoring and accurate detection of specific traits (e.g., fluctuations in animal interstitial fluid) to understand the physiological and behavioral changes occurring in animals. With reference to this, a WAIT4 (Welfare: Artificial Intelligence and new Technologies for Tracking Key Indicator Traits in Animals Facing Challenges of the Argo-ecological Transition) has been implemented with the aim to increase the technical possibilities to assess animal welfare status. A new sensor has been proposed to assess the kinetics of key physiological variables (Na^+^, K^+^, and pH) in animal interstitial fluid by microneedle patches, while the acquired data are to be analyzed and interpreted by machine learning algorithms in relation to animal welfare [166].

*Saliva.* The monitoring of lactate levels in animal saliva can be an indicator of stress level, as well as the health status of animals. Nowadays, microfluidic sensing systems based on materials such as carbon nanotubes and graphene have become popular among animal handlers and farm owners for enzymatic uric acid detection in saliva, detecting lactate variations, and fluoride detection [167].

#### 4.2.2. Transport and Handling

*Stress Monitoring During Transport*. Transport is a critical point within the meat chain where animal welfare can be compromised due to poor conditions related to factors such as watering, feeding, air flow, rest, load density, a high or low temperature, humidity, etc., in a vehicle transporting animals from farm to the livestock market and/or slaughterhouse. Nowadays, besides standard sensor solutions for the measurement of the heart rate and skin temperature of animals and the environmental conditions within transport vehicles, biosensors are widely used as stress indicators [167,168,169]. Salivary biomarkers are generally used to detect cortisol, which evaluates the hypothalamic–pituitary–adrenal axis, salivary alpha-amylase (sAA) [170], and CgA, which are related to the autonomous nervous system [171], and total esterase (TEA) and some of its components such as salivary lipase (sLip) and butyrylcholinesterase (BChE), which are enzymes that have been related to situations of pain and discomfort [172].

#### 4.2.3. Pre-Slaughter Welfare Assessment

Biosensors can assess the physical and emotional state of animals before they are slaughtered, as well as monitor the handling practices in slaughterhouses (the unloading of livestock after transport, stay in lairage/pens, and stunning procedure), ensuring that animals are treated humanely and in accordance with welfare standards to verify human handling practices. The application of biosensors may also contribute to ante mortem examination, a component of the meat inspection system, carried out by official veterinarians. Monitoring parameters like cortisol levels using biosensors in combination with heart rate can help to ensure that animals are calm and not experiencing unnecessary stress, which is important for both welfare and meat quality [104,173], while an increased body temperature may relate to the animal’s health condition. Such an application of biosensors may also add to a more automated ante mortem clinical examination of animals intended for slaughter and increase the relevance of meat inspection by providing accurate food chain information (FCI) data that can be used for risk assessment in the farm–slaughterhouse continuum.

#### 4.2.4. Application in Precision Livestock Farming

The application of biosensors in large animal populations in intensive commercial farming has excellent potential. Some biosensor applications within the precision livestock farming (PLF) system include the examples given below.

*Early Disease Detection.* Biosensors can be used to detect early signs of disease in large herds, allowing for timely interventions. For example, a proactive approach related to the application of biosensors for the detection of disease outbreaks through farm wastewater-based epidemiology (WBE) was recently proposed to collect comprehensive environmental and public health data to assist in timely health interventions during the COVID-19 outbreak [174]. Such an approach can also be a novel solution to monitor animal health on farms using wastewater discharge to detect the presence of disease-causative agents, which should allow for timely interventions either to maintain the health of food-producing animals or monitor fecally associated discharges of pathogens/AMR genes to prevent the cross-contamination of the environment. Another approach is the possibility of detecting animal infectious diseases and/or pathogens of high public health importance using nano-based biosensors [2].

In all, there is a good perspective associated with the use of biosensors in animal health and welfare monitoring within PLF systems, since they offer numerous advantages, such as non-invasive or minimally invasive approaches, providing real-time data for farmers and veterinarians, which are crucial for the early detection of animal health and welfare status and targeted interventions. The data acquired through regular applications of biosensors enable the continuous monitoring of selected health and welfare parameters of food-producing animals on farms and their integration within data management systems, allowing for automated health monitoring and analysis. The major challenges for the introduction of sensors into regular monitoring schemes for animal health are related to biosensors’ accuracy and calibration to provide reliable data, since they encompass animals wearing or being implanted with biosensors, as well as the costs and scalability to deploy them across large populations of animals.

#### 4.2.5. Slaughterhouse and Meat Processing

*Post-Slaughter Indicators.* Monitoring selected biochemical markers post-slaughter can provide information on whether animals were stressed or ill before slaughter, namely for animal welfare (e.g., stress hormones, such as Cortisol, and Cg A) and general animal health status (e.g., acute-phase proteins, such as haptoglobin, and Pig-Major Acute Proteins—MAP), which can be used to improve welfare practices and farm biosecurity [104].

*Pathogen detection.* Foodborne pathogens can enter the meat chain at various and multiple points along the F2F continuum, including during slaughter, processing, packaging, and distribution (Figure 2). Contamination with these pathogens poses serious health risks to consumers, including foodborne illnesses that can lead to severe outcomes or even fatalities. In the meat production chain, biosensors can be applied at various stages, from the prevalence of foodborne pathogens on farms and raw material inspection to the testing of the final product, enhancing the ability to quickly detect contamination and enabling timely responses (e.g., control measures and corrective actions). This helps in preventing outbreaks of foodborne illnesses and maintaining consumer trust in meat products. Biosensors operating with different transducing principles are being increasingly used for detecting foodborne pathogens and contaminants, ensuring food safety and public health. They can identify pathogens such as *Salmonella*, *E. coli*, *Listeria monocytogenes*, etc., and viruses by targeting specific proteins or DNA sequences [175,176,177,178,179,180]. Some examples include electrochemical biosensors leveraging antibody–antigen binding or aptamer-based approaches, which can achieve the highly sensitive detection of *Salmonella* or *E. coli* in various food matrices [181,182,183], as well as phagosensors biosensors that use bacteriophages as a bioreceptor to detect enteric bacteria in water samples, including *S. typhimurium* [184], biosensors for the detection of *S. typhimurium* in eggshells [185], and biosensors based on antimicrobial peptides as a bioreceptors for *Salmonella* detection by electrochemical impedance [186]. Different optical biosensors, including fluorescent and colorimetric, have been proposed as well, including aptasensors, which are fluorescently labeled to enable the detection of *E. coli* [187], antibody-based nano biosensors used to enable the highly sensitive detection of *E. coli* in food and water samples [188], and colorimetric sensors for food (milk) samples [189]. Further, a reusable sensor based on QCM technology with antifouling nanocoatings on the sensing surface for the accurate detection of *E. coli* in liquid (milk) or solid (hamburgers) food samples [190], FET platforms for the detection of Gram-positive and Gram-negative bacteria species, such as a multiplex detection biosensors based on an organic FET for the detection of two types of Gram-positive and -negative bacteria [191], a nanozyme-mediated MRS DNA biosensor for the rapid detection of *Listeria monocytogenes* in chicken meat [97], and many others can be found in the literature. Recently, a newly emerging technology based on CRISPR/Cas (clustered regularly interspaced short palindromic repeats and associated protein (Cas)) technology has received attention. CRISPR/Cas system-based biosensors appear to have an advanced flexibility, sensitivity, and specificity, enabling broad application and POC diagnosis in the field of environmental monitoring and food safety (the detection of foodborne pathogens). CRISPR/Cas is a novel biological tool, representing a variety of nucleic acid detection technologies (RNA and DNA) that have been successfully developed and applied in medical diagnosis, life sciences, and food safety. Such systems have a great potential, in particular, to enhance the detection of foodborne pathogenic bacteria in the meat chain, but also in aquaculture, e.g., *E, coli* O157:H7, *S. typhimuirum*, *S. enteritidis*, *L. monocytogenes*, methicillin-resistant *Staph. Aureus* (MRSA), and *Vibrio parahaemolyticus* (in aquaculture), with a detection time ranging from 15 min to 5 h. Different fluorescent, colorimetric, and electrochemical biosensors can be based on CRISPR/Cas technology. For example, electrochemical CRISPR/Cas-based biosensors showed a limit of detection (LOD) as low as 10 CFU/mL. A current limitation of this technology is that it is still in the stage of detecting only single foodborne pathogens, has complex working procedures related to nucleic acid extraction and amplification causing false-positive results, and cannot distinguish between live and dead bacteria. To address these challenges, new, fit-for-purpose aptamers should be deployed to enable the differentiation between live and dead bacteria, as well as the development of non-nucleic acid target detection such as metal ions, proteins, and adenosine triphosphate (ATP) [192].

*Chemical contaminants.* Antibiotics are widely used as bacteriostatic agents to combat microbial infection in animals and are almost unavoidable in the treatment of bacterial infections. Inappropriate antibiotic drug dosages, on the other hand, may result in antibiotic residues in livestock and poultry products (meat, milk, and eggs), aquatic products, and vegetables, resulting in a variety of side effects on human health. Antibiotic residues promote the spread of antibiotic-resistant bacteria, cause allergies like penicillin, and cause other severe pathologies such as cancers (oxytetracycline, sulfamethazine, and furazolidone), bone marrow toxicity, mutagenic effects, anaphylactic shock, reproductive disorders (chloramphenicol), and nephropathy (gentamicin). Biosensors are also used for the detection of chemical contaminants such as antibiotics [193,194], which is critical given the concern about antimicrobial resistance and to ensure that meat/meat products comply with regulatory antibiotic residue limits. Pesticides can accumulate in animal tissues and meat [195,196,197], as can growth hormones used to promote growth in livestock [198], dioxins [199], and sulfadiazine and acetaminophen [200], which can enter the meat chain through various environmental pathways, including contaminated feed and industrial pollution. Biosensors can additionally be used to detect the bioaccumulation of heavy metals such as lead, cadmium, and mercury, which can contaminate meat through environmental exposure [201], as well as natural toxins (mycotoxins) such as Aflatoxins, Ochratoxin A, Fumonisins, Zearalenone, and Trichothecenes, which can accumulate in tissues of food-producing animals due to the consumption of contaminated feed [202,203] and pose risks to consumers’ health.

*AMR detection.* In the era of increasing AMR related to pathogens of public health importance, including foodborne pathpgens, the rapid detection and monitoring of antibiotic resistance in the meat production chain is of the utmost importance to control and prevent AMR’s spread, particularly in international trade. Current methods allow for the separate detection of either selected pathogen via traditional (culturing), immunoassay (ELISA), and molecular methods (PCR) or antibiotic resistance genes via molecular methods such as PCR, DNA microarray, whole-genome sequencing (WGS), and metagenomics [204], thus requiring multiple assays. Therefore, there is a need for POC devices that can simultaneously detect pathogens (infectious agent) and their antibiotic resistance at the given module along the meat chain. For example, in 2008, an optical, silicon-based biosensor was developed to detect the *tuf* gene in blood cultures for the identification of the *Staphylococcus* genus, the *femB* gene for the identification of *S. aureus* species, and the *mecA* gene for the identification of methicillin resistance with an LOD = 5 × 10^7^ CFU/mL [205], as were an electrochemical sensor to be used in bacterial cultures combining a new class of non-biological binder molecules with electrochemical impedance spectroscopy (EIS)-based sensor detection for the detection of Gram-positive bacteria [206] and an integrated, dual-channel electrochemical biosensor for the detection of Enteropathogenic (EPEC) *E. coli* based on monoclonal antibodies against a virulence marker (*EspB*) and markers for AMR detection (β-lactam resistance marker and β-lactamase) developed in [207]. The integration of biosensors can have a profoundly beneficial impact on tackling one of the most important global public health challenges, antimicrobial resistance, and should be conducted within the One Health context [208] to cover the environment–animal–human ecological compartments.

### 4.3. Meat Quality Traits

Biosensors are being increasingly used for the rapid and accurate detection of the most important meat quality traits, such as freshness, sensory and nutritional attributes, and the spoilage of meat and meat products. Some examples of biosensors’ applications are given below.

*Freshness and spoilage.* Biosensors can measure the freshness and/or spoilage of meat/meat products by detecting the levels of certain compounds typically associated with the degradation of meat proteins and lipids, including purine derivatives, such as hypoxanthine and xanthine [209], ammonia [210], trimethylamine [211], and hydrogen sulfide [212] and food spoilage biomarkers such as volatile organic gases, microorganisms, and enzymes [213].

*Sensory and nutritional attributes.* Keeping meat fresh on the journey from the slaughterhouse to the consumer is not always possible, and, therefore, the development of easy-to-use POC devices for meat freshness can help consumers to buy meat as fresh as possible. Some biosensors assess meat quality traits related to sensory characteristics and nutritional value. For example, biosensors can detect visual texture, color, visible fat and natural drip in raw meat [214], and aroma and flavor in thermally processed meat [215] by detecting changes in specific proteins or metabolites. Some biosensors can evaluate freeze/thaw cycles for the detection of hemin in beef samples [216]. A series of graphene FET-based electronic noses have been developed so far for in-field and rapid food freshness evaluations under refrigerator and room-temperature conditions for the detection and quantification of olfactory compounds [217,218,219].

### 4.4. Food Fraud and Food Crime

Food fraud is a widespread global concern and is also frequently associated with meat products. Biosensors are becoming increasingly valuable in combating food fraud and food crime in the food (meat) production chain. Their ability to provide precise, real-time, and reliable data helps to ensure the authenticity and safety of meat products. Some examples of biosensors’ applications to address these issues are given below.

*Detection of Adulteration.* Biosensors are used to detect chemical adulterants, and this is related to unauthorized chemicals or food additives or packaging materials that may be used to alter the appearance or quality of meat [220,221]. A specifically functionalized graphene derivative was employed for the development of an impedimetric genosensor for pork adulteration in real meat samples with a determination limit of detection of 9% W/W for pork content in beef [222], and an MIP nano gel-based sensor was proposed for the detection of pork contamination in real beef extract samples, showing an LOD of 12 µg/mL [89]. These sensors can also identify specific chemical markers or residues indicative of fraud.

*Species and origin authentication.* Biosensors can verify the species of meat, helping to prevent fraud where cheaper or different species are substituted for higher-value meats, for example, when beef products should be free from pork residues due to religious or cultural reasons. For example, label-free impedimetric genosensors for the sensitive detection of pork residues in meat, based on single-stranded DNA probes specific for the pork mitochondrial genome, have been developed. Such biosensors enable the detection of pork residues in beef in less than 45 min (including sample preparation), with an LOD of 9% w/w pork content in beef samples [220]. Spectroscopy-based sensors were developed to detect frequent fraudulent practices related to minced meat substitution with cheaper raw materials, such as beef with bovine offal and pork with chicken [223]. Similar sensors might be also used for the detection of the authenticity of high-value products such as Wagyu or Kobe beef. In the global market, many meat products are officially protected bearing the mark ‘Protected Designation of Origin’ or ‘Protected Geographical Indications’. Such products typically have a higher market value and because of this, are more vulnerable to fraudulent practices associated with the use of cheaper raw materials from other species or geographical regions. DNA-based biosensors can accurately identify meat species by analyzing genetic material and can be proactive and cost-effective approach to ensure food authenticity and verify food origin [29]. This is also because DNA is more stable and can withstand harsh environmental conditions in comparison with proteins and metabolites, thus being an excellent solution as a bioreceptor for the quantitative detection of food fraud and adulteration [224,225].

Biosensors can be used in combination with other traceability technologies to verify the origin of meat products. For example, sensors embedded in packaging or labels can track and authenticate the meat’s journey from the farm to the table, ensuring that claims about its origin are accurate, thus contributing to smarter food traceability [35].

*Consumer protection.* Portable biosensors can allow for on-site testing to determine the quality and authenticity of meat products in retail settings or at home, such as the verification of product labels or packaging to check claims made about the meat’s origin, quality, or content [225]. This adds a layer of transparency and trust, empowers consumers to make informed choices, and reduces the risk of being exposed to fraudulent practices.

In all, the deployment of biosensors in detecting meat quality traits can be an asset for competent authorities (inspection) verifying the compliance of meat products with food quality regulations, as well as facilitating a quicker response to potential food fraud. Consumers may also benefit from using biosensors to ensure they receive the product they are paying for, thus reducing the risk of fraudulent labeling.

### 4.5. Risk-Based Meat Safety Assurance System

Biosensors can become an integral part of the risk-based meat safety assurance system (RB-MSAS), providing the real-time monitoring of selected foodborne hazards and ensuring meat safety throughout the production chain. They should be also included in the future training of official veterinarians to monitor the RB-MSAS [226,227]. An overview of the application of biosensors in the meat production chain is presented in Table 1.

**Figure 4:**
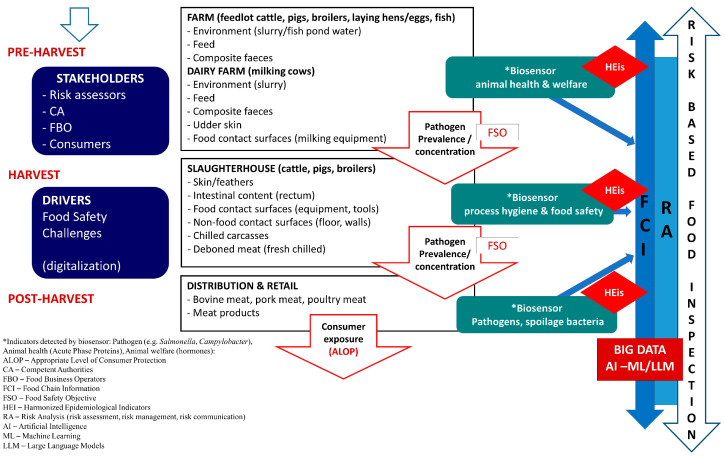
A model for field implementation of multiplex, point-of-care biosensor in farm-to-slaughterhouse continuum [227].

Their contribution to the RB-MSAS is important, since they enable the integration of data acquired along all modules in the meat chain (farm–slaughterhouse–meat processing–distribution–retail continuum) via IoT, such as a blockchain approach [227]. Namely, they allow the integration of data from various stages of production and inspection into a centralized system for the comprehensive monitoring of meat safety, allowing a holistic approach. For example, biosensors contribute to the FCI dataset by providing real-time information on foodborne hazards and facilitating FCI flow from farm to slaughterhouse–meat processing–retail (bottom-up) and backward, from retail to farm (top-down). This also enables record keeping and the tracking of meat products from production to the point of sale within the blockchain system [35]. This may help to maintain comprehensive records of safety and quality checks throughout the supply chain, analyze trends, and conduct risk assessments using AI and ML. It is also important to enable interoperability to ensure that the acquired data are compatible with existing food chain information systems, which should facilitate seamless integration and enhance the overall effectiveness of MSAS (Figure 4). Further, the important contributions of biosensors’ application within MSAS are related to epidemiological monitoring and surveillance by providing accurate and timely information on potential foodborne hazards in various modules along the meat chain, including outbreaks, thus enabling comparative analyses of meat safety data from various sources via Harmonized Epidemiological Indicators (HEIs) [227,228]. Another advantage of biosensors within the MSAS is the capability to automatically generate reports for regulatory compliance, documenting adherence to food safety regulations. By aligning biosensor data with HEIs, competent authorities can perform better risk assessments and manage the risks associated with meat products, leading to more effective interventions and public health protection. This helps competent authorities in risk management decisions and allocating resources more effectively, focusing on the most critical points in the production chain. Parallel to that, it also contributes to increasing consumer confidence in food control systems, providing a better transparency. Overall, by integrating biosensor data into a comprehensive RB-MSAS, the meat industry can achieve better risk management, operational efficiency, and build consumers’ trust in the safety of meat/meat products.

### 4.6. Opportunities and Challenges for Biosensors’ Application in the Meat Production Chain

**Figure 5:**
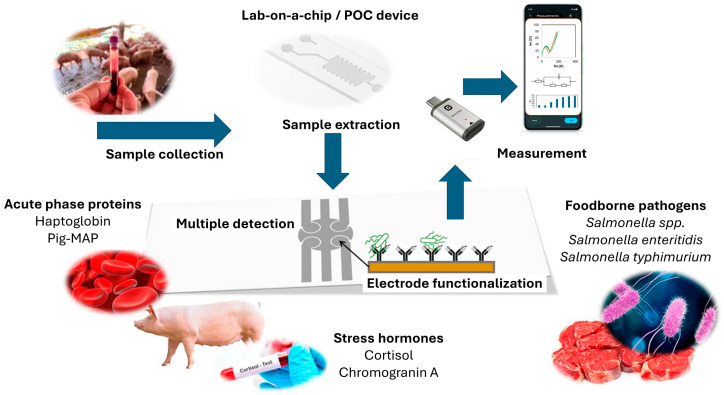
A structure model of biosensor for application in the meat chain.

Due to the complexity of the meat supply chain, frequently associated with long storage and distribution/transport periods for meat/meat products based on the specific requirements of retail chains, biosensors are needed to enable proper real-time monitoring in all phases of production [28]. As discussed above, biosensors can enhance food safety, quality control, and traceability in the food chain. The opportunities for future applications of biosensors along the meat chain (Figure 5) are numerous and they relate to improved food safety monitoring, quality control and shelf-life monitoring, traceability, authenticity, and real-time production process control [229]. 

*Improved food safety monitoring*. As discussed, biosensors offer the rapid, on-site detection of pathogenic microorganisms such as *Salmonella*, *Escherichia coli*, *Listeria monocytogenes*, and *Campylobacter*, which are common in meat production. Biosensors can detect these foodborne pathogens at low levels before contamination spreads through the supply chain, thus allowing for timely actions to prevent the further transfer of pathogens along the meat chain [230].

*Quality control and shelf-life monitoring.* Biosensors can track the freshness of meat by detecting VOCs or pH changes associated with spoilage, which are crucial for ensuring product quality [213,214].

*Traceability and authenticity.* Biosensors enable the detection of DNA or protein markers specific to animal species, which helps in verifying meat authenticity, thus preventing fraud (e.g., mislabeling or adulteration) [29].

*Real-time production process control.* The integration of biosensors in automated systems allows for continuous monitoring along all modules in the meat chain [38], such as at the farm (animal welfare and health), during slaughtering and meat cutting (microbial process hygiene), and during packaging processes (control of cross-contamination), enhancing the efficiency of quality control and reducing human error.

Challenges to the regular application of biosensors are related to several gaps that may limit their widespread adoption and effectiveness. Identifying these gaps is crucial for guiding further research and development. Some examples are given below.

*Limited Range of Detection.* Many biosensors are designed to detect specific pathogens, contaminants, or spoilage markers, but the range of their detection is often narrow and does not correspond either with regulatory limits or recommended values according to best practices. Therefore, the issues related to detection range (an appropriate level of detection, not always related to the lowest possible limits) should correspond with food regulations and market requirements and allow for a balanced approach in the technological development of biosensors [231].

*Sensitivity and Specificity.* This issue is related to false positives or negatives. The specificity of biosensors can be compromised by cross-reactivity, leading to false positives (detecting a hazard where none exists) or false negatives (failing to detect an actual hazard) [229]. This is particularly an issue in complex samples within the meat production chain (e.g., feces, slurry, carcass, meat juice, and meat products) which contain dirt, proteins, fats, and other compounds that can interfere with a biosensor’s ability to detect specific analytes. Overcoming these interferences is essential for reliable, accurate measurements in real-world applications.

*Lack of Standardization and Calibration*. Biosensors can suffer from variability in their performance due to differences in calibration, environmental conditions, or the biological materials used. This lack of standardization can lead to inconsistent results and limit the reliability of biosensors in diverse settings [33].

*Environmental and operational conditions*. Since meat production environments (farms, slaughterhouses, processing plants, etc.) are subject to extreme conditions like fluctuating temperatures, humidity, and the presence of organic matter, these factors can affect the performance and durability of biosensors, limiting their usability in harsh operational settings.

*Regulatory Compliance and Auditing.* Biosensors, particularly those used in food safety and quality control, face strict regulatory frameworks that vary across regions, such as the European Food Safety Authority (EFSA) and European Medicine Agency (EMA) in the European Union or Food and Drug Administration (FDA) regulations in the United States. The adoption of new biosensor technologies requires adherence to these regulations and approval based on long-term validation data. This can delay the adoption of innovative biosensors in the meat industry, which can delay their commercialization and widespread use [231].

*High costs and commercialization issues*. The major challenges are related to costs and economic feasibility, especially for those biosensors with advanced capabilities, which remains a barrier to their widespread adoption, particularly for small- and medium-sized enterprises (SMEs) in the meat industry. Another concern is the potential for commercial applications of biosensors with regard to complexity (integration with existing meat safety inspection systems, such as the MSAS, which can be complex and require training) and accuracy (calibration to avoid interference from other substances and ensure accurate results). Namely, the integration of sensing systems with the MSAS may require significant and costly modifications to equipment or workflows to enable data management and their interpretation. Therefore, ensuring that biosensor technology is affordable is crucial for its widespread adoption and regular use.

*Ethical considerations.* The primary issue is to ensure that biosensor devices do not to cause any discomfort or harm to animals. Other ethical aspects relate to concerns around animal welfare, data privacy, economic equity, environmental impact, consumer trust, labor dynamics, and regulatory oversight to ensure that this technology contributes positively to the meat industry and society as a whole.

In all, biosensors in the meat continuum chain face several limitations, including a low sensitivity, limited specificity, and potential interference from complex matrices. While numerous solutions have been proposed in the scientific literature, most are still the subject of research due to the complexity of practical implementation or their high mass production cost. Other issues are related to the complexity of in-field sample extraction and preparation, which is not practical for average end-users. Additionally, real-time applications are hindered by regulatory challenges and the need for user-friendly designs that can be easily integrated into industrial workflows. The adoption of new biosensor technologies requires adherence to these regulations and approval based on long-term validation data. This can delay the adoption of innovative biosensors in the meat industry, commercialization, and widespread use.

Therefore, future research should focus on fit-for-purpose advanced fabrication methods, such as nanomaterial-based designs and nano- and micro-fabrication technology to develop a low-cost device with an enhanced sensitivity and performance. Regarding sample preparation protocols for in-field use, the future trend will be focused on developing multiplex platforms for the simultaneous detection of several biomolecules relevant to animal health, welfare, food safety, and quality and their integration into autonomous PoC and LoC devices, with AI protocols for data analysis. Improving sensor selectivity through molecular engineering and AI-driven data analysis will enable the more accurate and reliable detection of contaminants in the meat production chain. However, the market demands a cheap and reliable product, so future efforts will be on the development of scalable, low-cost manufacturing techniques, such as roll-to-roll printing and 3D bioprinting to facilitate mass production while maintaining a high performance, affordability, and cost-effectiveness.

## 5. Conclusions

The application of biosensors in the meat production chain represents an advancement that may contribute to food system transformation by enhancing animal health and welfare, food safety and food quality control, reducing climate change’s impact, increasing consumers’ confidence in meat business operators, and fostering transparency regarding data management. Biosensors, as POC devices capable of monitoring biomarkers specific to animal health (acute-phase proteins) and welfare (stress hormones), microbial contamination (foodborne pathogens, e.g., *Salmonella*, *Campylobacter*, Shiga-toxin-producing *E. coli*/STEC, and *L. monocytogenes*), AMR (resistance genes), and environmental contaminants (pesticides, dioxins, and mycotoxins), have the potential to address several longstanding challenges in the meat industry. One of the key benefits of biosensors is their ability to improve food safety by the rapid quantitative detection of foodborne pathogens and contaminants along modules in the meat production chain (farm–slaughterhouse–meat processing–retail continuum) in real time. This not only minimizes the risk of occurrence of foodborne illnesses, but also strengthens consumer confidence in the meat supply chain. Additionally, by monitoring the health and well-being of livestock, biosensors can contribute to higher farm biosecurity and welfare standards and more efficient production practices. Furthermore, meat supply chain transparency will be enhanced, as biosensors provide traceable data that may be deposited in open-access platforms, allowing consumers to make informed choices. However, the deployment of biosensors also raises important ethical questions, such as ensuring that biosensors do not cause any discomfort or harm to animals, as well as enabling certain level of data privacy on animal health, welfare, and food safety. Challenges also include maintaining economic equity related to a certain meat business operator. For example, small-scale farmers and meat business operators may face barriers to accessing this technology due to high costs, risking further inequalities in the industry. Therefore, initiatives to subsidize or make biosensors more affordable to all stakeholders involved in the meat production chain are crucial. In addition, automation in animal health, welfare, and food safety monitoring and data-driven processes for analyses and risk assessment based on AI and large language models (LLMs) could displace workers, necessitating retraining programs to support the affected individuals. Further, respect for traditional knowledge and practices is crucial in balancing innovation with cultural heritage and preserving traditional production practices characteristic for certain geographical regions and national habits. Environmental considerations also play a significant role. While biosensors can optimize resource use (e.g., optimized animal nutrition and health) and reduce waste, attention should be also given to the production and disposal of these devices, which must be managed responsibly to prevent ecological harm. Using sustainable materials in biosensors’ manufacturing and recycling programs can mitigate such impacts. Additional efforts should be made in terms of integrating the data acquired by biosensors within RB-MSAS to contribute to FCI flow and HEIs and enhance an integrated approach toward meat safety. This will improve consumer confidence in livestock raising conditions and food control systems, as well as foster informed purchasing decisions. The vast amounts of information generated by biosensors require robust governance frameworks to ensure fair usage and protect key stakeholders’ rights (industry, competent authorities, researchers, and consumers). To realize the full potential of biosensors’ applications in the meat chain, collaborative efforts among key stakeholders will be essential. Further research should be conducted to address not only technical aspects in biosensors’ manufacturing (nanomaterials, detection methods, and sensitivity) and environmental protection, but also to develop a model system for their application to achieve regulatory approval. Additional efforts should be made to find appropriate solutions regarding socioeconomic aspects related to biosensors’ affordability and inclusivity for small-scale producers. These research efforts should ideally be conducted through a trans-disciplinary collaboration between life and social sciences/humanities to achieve acceptable and fit-for-purpose solutions enabling a more efficient and sustainable meat industry.

## Figures and Tables

**Table 1:** Biosensors in the meat production chain.

Biosensors	Applicability in the Meat Production Chain				Reference
	F	S	MP	R	
Stress detection sensors (hormones)	x				[2,166]
Breath sensors (VOCs for the detection of metabolic and pathological processes)	x				
Perspiration sensors (metabolites in sweat)	x				
Tears sensors (glucose monitoring)	x				
Salivary sensors (uric acid—metabolic syndrome, renal syndrome, and abnormalities in purine metabolism)	x				
Progesterone sensors (the detection of ovulation)	x				
Food and feed sensors (dietary inputs/nutrition, bioactive compounds, and microbiological and chemical contamination)	x				
Infectious disease detection sensors (BRD, AIV, FMD, BVD, PRRSV, avian influenza, and mastitis)	x				
Sensor for continuous glucose monitoring (CGM) in dairy calves	x				[100]
Sensor for monitoring of stress and animal welfare (hormonal levels) in pigs’ blood	x	x			[103]
Glucocorticoid sensor (saliva, excreta, fecal samples, milk, hair/feathers, and eggs)	x				[104]
Sensors for inflammatory markers (acute-phase proteins)	x	x			[5,103,113,117,118,120]
Sensors for metabolic markers (breath, blood, faeces, skin, urine, and vaginal fluids)	x	x			[121,122,123,124,125,126,127,128,129]
Sensors for infectious disease detection: Bovine. BRD (serum), BVD (serum; LOD of 103 CCID/mL), EBL, FMD, bovine mycoplasma, mastitis (Hp in serum), and Campylobacter in dairy cattle (LOD of 3 CFU/mL)	x				[126,130,134]
Pigs. AFSV, PRRSV, SIV, and PCVAD	x				[138,139,142,143]
Poultry. H5N1 (LOD of 10^3^ EID50/mL), *Clostridium perfringens* (LOD of 0.26–0.27 lg/CFU), audio-based sensor detection system (Newcastle Disease, Infectious Bronchitis, Infectious Laryngotracheitis, AI, MG, CRD, infectious sinusitis, mycoplasmosis), antibiotic residues	x				[114,145,146,147]
Early disease detection * WBE	x				[174]
Sensors for monitoring reproductive health Hormonal levels (progesterone and estrogen in milk, urine, and feces) in dairy herds	x				[149,150,151]
Estrus detection	x				[160,161,162]
Sperm quality (in livestock)	x				[163,164,165]
Animal welfare monitoring sensors Behavioral, physiological, and nutrition status Animal Intersticial Fluid (ISF)	x				[165]
Saliva	x				[167,170,171]
Transport and Handling Stress Monitoring	x	x			[166,167,168,169,170,171,172]
Pre-slaughter welfare assessment		x			[104,173]
Slaughterhouse and meat processing					
Post-slaughter indicators (animal health and animal welfare)		x			[103]
			x	x	[97,175,176,177,178,179,180,181,182,183,184,185,186,187,188,189,190,191]
Pathogen detection (*Salmonella*, *Campylobacter*, *Shiga-toxin-producing E. coli (STEC)*, *Listeria monocytogenes,* and *viruses*)		x			[192,193]
Antibiotic residues	x	x	x	x	[194,195,196]
AMR		x			[197]
Pesticides Growth promoters Dioxins		x x x			[198]
Mycotoxins			x	x	[201,202]
Meat quality traits Freshness and spoilage (purine derivatives, ammonia, and VOCs)		x	x	x	[208,209,210,211,212]
Sensory and nutritional attributes (color, texture, visible fat, aroma, and flavor)			x	x	[213,214,215,216,217,218]
Food fraud Food adulteration		x	x	x	[219,220,221]
Species and origin authentication			x	x	[222,223,224]

F: farm; S: slaughterhouse; MP: meat processing; R: retail; LOD: limit of detection; BRD, bovine respiratory disease; BVD, bovine viral diarrhea; EBL, Enzootic Bovine Leucosis; FMD, Foot and Mouth Disease; Hp, haptoglobin; BSE, Bovine Spongiform Encephalopathy; AFSW, African Swine Fever; AI, avian influenza; PRRSV, porcine reproductive and respiratory syndrome virus; SIV, Swine Influenza Virus A; PCVAD, porcine circovirus-associated disease; MG, *Mycoplasma* gallisepticum; CRD, Chronic Respiratory Disease; WBE, wastewater-based epidemiology; * Potential for application in livestock; farming systems; AMR: antimicrobial resistance; VOCs: volatile organic compounds.

## Data Availability

No new data were created or analyzed in this study. Data sharing is not applicable to this article.

## Abbreviations

The following abbreviations are used in this manuscript:

AI	Artificial intelligence
AMR	Antimicrobial resistance
APP	Acute-phase proteins
CFU	Colony-forming units
COVID-19	Corona Virus Disease of 2019
EC	Electrochemical
EIS	Electrochemical impedance spectroscopy
ELISA	Enzyme-linked Immunosorbent Assay
F2F	Farm-to-fork
FCI	Food chain information
FET	Field-effect transistor
IoT	Internet of Things
HEIs	Harmonized epidemiological indicators
HPLC	Reaction—PCR, High-Performance Liquid Chromatography
LC-MS	Liquid chromatography–mass spectrometry
LC-UV	Liquid chromatography and UV detector
LED	Light-emitting diode
LOC	Lab-on-a-chip
LOD	Limit of detection
MIPs	Molecularly Imprinted Polymers
ML	Machine learning
MRS	Magnetic Relaxation Switching
POC	Point-of-care
PCR	Polymerase Chain Reaction
PLF	Precision livestock farming
PMB	Portable multifunction biosensor
PIC	Photonic integrated circuit
QCM	Quartz crystal microbalance (resonator)
RPA	Recombinase Polymerase Amplification
RB-MSAS	Risk-based meat safety assurance system
SAW	Surface acoustic wave
SERS	Surface-Enhanced Raman Spectroscopy
SPR	Surface plasmon resonance
VOCs	Volatile organic compounds
